# End-to-End Model-Based Detection of Infants with Autism Spectrum Disorder Using a Pretrained Model

**DOI:** 10.3390/s23010202

**Published:** 2022-12-25

**Authors:** Jung Hyuk Lee, Geon Woo Lee, Guiyoung Bong, Hee Jeong Yoo, Hong Kook Kim

**Affiliations:** 1 School of Electrical Engineering and Computer Science, Gwangju Institute of Science and Technology, Gwangju 61005, Republic of Korea; 2 AI Graduate School, Gwangju Institute of Science and Technology, Gwangju 61005, Republic of Korea; 3 Department of Psychiatry, Seoul National University Bundang Hospital, Seongnam 13620, Republic of Korea; 4 College of Medicine, Seoul National University, Seoul 03980, Republic of Korea

**Keywords:** autism spectrum disorder, end-to-end neural network, pretrained model, joint optimization, autoencoder, bidirectional long short-term memory (BLSTM)

## Abstract

In this paper, we propose an end-to-end (E2E) neural network model to detect autism spectrum disorder (ASD) from children’s voices without explicitly extracting the deterministic features. In order to obtain the decisions for discriminating between the voices of children with ASD and those with typical development (TD), we combined two different feature-extraction models and a bidirectional long short-term memory (BLSTM)-based classifier to obtain the ASD/TD classification in the form of probability. We realized one of the feature extractors as the bottleneck feature from an autoencoder using the extended version of the Geneva minimalistic acoustic parameter set (eGeMAPS) input. The other feature extractor is the context vector from a pretrained wav2vec2.0-based model directly applied to the waveform input. In addition, we optimized the E2E models in two different ways: (1) fine-tuning and (2) joint optimization. To evaluate the performance of the proposed E2E models, we prepared two datasets from video recordings of ASD diagnoses collected between 2016 and 2018 at Seoul National University Bundang Hospital (SNUBH), and between 2019 and 2021 at a Living Lab. According to the experimental results, the proposed wav2vec2.0-based E2E model with joint optimization achieved significant improvements in the accuracy and unweighted average recall, from 64.74% to 71.66% and from 65.04% to 70.81%, respectively, compared with a conventional model using autoencoder-based BLSTM and the deterministic features of the eGeMAPS.

## 1. Introduction

Autism spectrum disorder (ASD) is a neurodevelopmental disorder that is characterized by difficulties in social interactions [1]. The prevalence of ASD has gradually increased, and it is currently a critical concern [2]. Early intervention is the key to improving the social skills of children with ASD [3]. In several studies, researchers have attempted to distinguish the unique vocal characteristics of children with ASD from those of children with typical development (TD). The feasibility of automatic screening for ASD based on the characteristics of children’s speech must be directly or indirectly determined. Earlier efforts have focused on the atypical speech patterns in children with ASD, such as pronunciations with distinctive patterns of vowels or acoustic features, such as pitch, the long-term average spectrum, or averaged intensity [4,5].

Using advanced machine-learning-based techniques for classification, in the current attempts to identify children with ASD, researchers focus on using features associated with abnormal patterns in the voices of children with ASD, or on using transformed features to explore the differences between children with ASD and TD children [6,7,8]. In these approaches, researchers typically use the predefined features from speech and audio signal processing. These features could be statistical features, paralinguistic features, such as those in the extended version of the Geneva minimalistic acoustic parameter set (eGeMAPS) [9,10,11], or spectral features obtained from a short-time Fourier transform [12,13]. As classification algorithms, supervised learning models, such as random forests, support vector machines (SVMs), *k*-nearest neighbors, and probabilistic neural networks [7,10,13,14], have achieved satisfactory performances in classifying children with ASD and TD.

While these approaches based on predefined features have promising outcomes, the validity of the features is still questionable because the acoustical features have not yet been proven as suitable biomarkers of autistic voices [5]. Although the feature extraction is the process of representing the characteristics of autistic voices, the mentioned classifiers do not consider this process because they are only trained using the predefined features and not at learning the process that is required to capture vocal characteristics.

In our previous work, we proposed an autoencoder (AE)-based feature-extraction model that can be used to extract the latent representations of the predefined features and then then fine-tune the classifier using the latent features from the AE model to alleviate this intermediate problem [15]. The AE-based feature-extraction model provides better results than those of models that use predefined features. Nevertheless, classification using the latent features has certain limitations because the latent features of the ASD and TD voices from the AE model have overlapping distributions with the features obtained from the eGeMAPS, which we investigated using *t*-stochastic neighbor embedding (t-SNE) analysis. While the feature-extraction AE is guided by the auxiliary task of ASD/TD classification using a small weight factor and is then fine-tuned, the entire model has a modular structure that does not allow the feature-extraction process to be jointly trained with the classification layers.

Therefore, we considered two distinct approaches to better represent the distinctive features of the voices of children with ASD. In the first approach, we replace the modular structure with a jointly optimized model. By doing so, the latent feature extraction is integrated into a single model to mitigate the ambiguity in the modular structure between the feature extractor and classifier. In the second approach, we replace the predefined features with those obtained from a pretrained neural network. The feature extractor is first combined with a classifier. Then, the combined model is fine-tuned using a training dataset of the ASD classification task. This second approach is an end-to-end (E2E) model that learns a complete task from inputs without intermediate modules or pipelines [16,17,18]. We can construct this model to execute classification tasks from the speech signal itself.

Thus, we propose an E2E model for detecting ASD from children’s voices based on the second approach. Specifically, the proposed E2E model comprises a wav2vec2.0-based feature-extraction model [19] and binary classifier that consists of two fully connected (FC) layers. The E2E model is fine-tuned using the ASD dataset collected from the subjects in the clinical and living rooms operated by psychiatrists. To compare the performance of the proposed E2E model, we trained the AE-based models using the same data with the extracted eGeMAPS features. As a modular structure, we trained the AE feature extractor using the eGeMAPS, and we fine-tuned the bidirectional long short-term memory (BLSTM)-based classifier [20,21] using AE-based latent representations, which we refer to as the AE-BLSTM model. In addition, we jointly trained the AE-BLSTM model per the first approach described above.

We organize the remainder of this paper as follows. In Section 2, we describe the specifications of the participant data, data processing, and feature extraction. In Section 3, we explain the network architecture and training methods of the AE-BLSTM model. In Section 4, we propose an E2E model for the detection of ASD from children’s voices. In Section 5, we evaluate the performance of the proposed E2E model and compare it with those of an AE-BLSTM model with and without joint optimization. Finally, in Section 6, we present the conclusions.

## 2. Data Collection for Autism Spectrum Disorder Classification

In this study, we used two datasets with audio data from the video recordings of ASD diagnoses collected between 2016 and 2018 at Seoul National University Bundang Hospital (SNUBH), and between 2019 and 2021 at a Living Lab. The Institutional Review Board (IRB) at SNUBH approved the use of fully anonymized clinical data for the retrospective analysis (IRB no. B-1909/567-110) in the existing research (IRB no. B-2003-603-301).

We collected the recordings in one of two typical clinic rooms in SNUBH, or in a room in the Living Lab. The clinic rooms in SNUBH are 365 cm × 400 cm × 270 cm and 350 cm × 350 cm × 270 cm, and the hospital noise level was approximately 40 dB. The dimensions of the Living Lab room are 350 cm × 190 cm × 270 cm, and the room is covered with soundproof material; thus, the room noise level was also around 40 dB. In particular, the Living Lab is a facility that was established to more effectively gather data for clinical procedures, and the room is divided into two spaces. One space is a playroom where children can be screened for ASD. In this playroom, we installed one monitor for visual excitation and seven Azure Kinect Developer Kits (DKs) for recording the audiovisual data. The Azure Kinect DK comprises a red–green–blue (RGB) camera, depth camera, and hexagonal microphone array. The hexagonal microphone array consists of seven microphones: six are placed at every hexagonal edge, and one is located at the center of the hexagonal array. In this work, seven Kinect DKs are used together to capture video of the subject in a room in any direction; one of the seven Kinect DKs, located at the front of the room, is used for recording the speech signals of the subject. In particular, the speech signals from the center microphone are used for ASD detection. The second space is the server room, in which the recorded data are stored on a massive storage server. The server separately stores video and audio data from each camera and the microphone array, respectively. We collected audio files with the high intelligibility of children’s voice.

Consequently, we collected the audio files of 191 children who we assessed using seven instruments, and we based the final diagnosis on the best clinical estimate diagnosis according to the *Diagnostic and Statistical Manual of Mental Disorders, Fifth Edition* (*DSM-5*) [22] ASD criteria and a licensed child psychiatrist using all the available participant information [23]. We labeled each audio file with the visiting date, a unique ID number, the diagnostic protocol, and the gender, age, and diagnostic result (ASD or TD). We arranged the participants’ ages per the behavior development screening for toddlers: (BeDevel)-Play (BeDevel-P) and BeDevel-Interview (BeDevel-I) [24], which were developed to diagnose Korean children between the ages of 9 and 42 months. The average age of the 191 children in this study was 27.39 months, with a standard deviation (SD) of 9.11 months. We noted the age when each subject visited the hospital for an examination. Among the children, 126 were diagnosed with ASD (78 males and 25 females), the average age of which was 32.20 months, with an SD of 6.83 months. The remaining 65 participants were children with TD (48 males and 40 females). We present the collected data distribution, means, and SDs for the ages in Table 1.

A doctor or clinician and the parents continually stimulated the children to induce socialized reactions based on the screening protocols; thus, the audio file recorded in this environment comprised speech signals that belonged to the children and attending adults. In addition to speech signals, various sound signals were simultaneously recorded, such as sounds from playthings, clattering, and dragging noises. To classify ASD only from children’s voices, we manually detected the intervals that corresponded to them, and we then stored the speech signals in each interval into a file. Furthermore, we split each file into a sequence of segments with lengths of 1 s, and we performed zero-padding for the last segment of the file if its length was shorter than 1 s for the training and evaluation of the ASD/TD detection models. We resampled each stored speech file from 16 kHz to 48 kHz with a monochannel format.

## 3. Conventional Autoencoder-Based Classification for Autism Spectrum Disorder

In this section, we review the conventional modular structure for classifying children with ASD and TD. In particular, we review a sequential training approach with an AE-based feature extractor using the eGeMAPS, followed by a BLSTM-based classifier with a pooling layer, as proposed in [23]. Then, we explain the fine-tuning approach for training the joint model of the AE-based feature extraction and BLSTM-based classifier.

An AE has the form of an encoder and decoder with a symmetric structure. The encoder projects each piece of input data into a low-dimensional latent space, and the decoder reconstructs the original data from the compressed latent features [24]. With the dimensionality reduction in the AE, the latent space embedding has a lower dimension than the input data. Thus, the latent feature, which is the so-called bottleneck feature, can represent the distinctive characteristics of the higher-dimensional input data [25].

**Figure 1:**
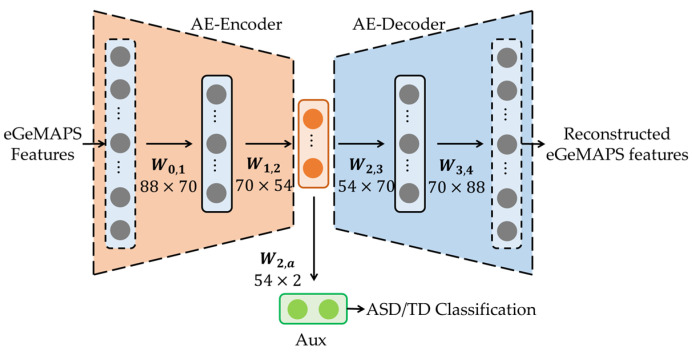
Network architecture for extracting bottleneck features using autoencoder with multi-task learning strategy.

We present an AE model that extracts the bottleneck features from 88-dimensional eGeMAPS features to classify children with ASD and TD in Figure 1 [23]. As depicted in the figure, the AE model consists of an FC-layer-based encoder and decoder. The encoder of the AE model comprises two FC layers with weight matrix dimensions of (88, 70) and (70, 54), and the decoder has a reverse structure to the encoder (i.e., two FC layers with weight matrix dimensions of (54, 70) and (70, 88)). The dimensions of the first FC layer are identical to the dimensions of the eGeMAPS features. An FC layer with (54, 2) dimensions is constructed to apply a multi-task learning strategy. In other words, the bottleneck feature of the AE model is used as the input to this FC layer, and the output is the binary label of ASD or TD, corresponding to the input data of the AE model. The auxiliary task of classifying the latent representations with binary labels of ASD or TD is intended to guide the bottleneck features from each class with separate distributions. To train the AE model using the auxiliary task of ASD/TD classification, we used the combination of the reconstruction loss ($L_{recon}$) of the AE and the classification loss ($L_{clsf}$) of the auxiliary task, as follows:

(1) $$L=\alpha \cdot L_{recon}+\left(1-\alpha \right)\cdot L_{clsf}$$

where α is the control parameter used to provide different emphases to the main and auxiliary tasks, which the authors of [23] set to 0.9.

**Figure 2:**
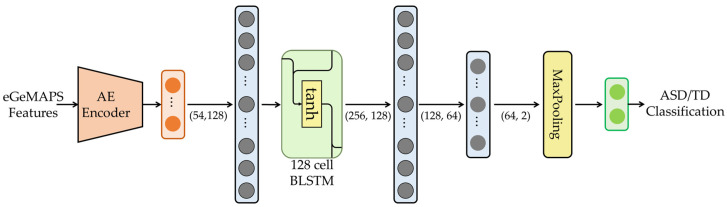
Network architecture for AE-BLSTM-based classifier for ASD/TD classification for fine-tuning BLSTM classifier of AE encoder and BLSTM classifier, which we refer to as AE-BLSTM-FT.

To perform the ASD and TD classification, we designed a BLSTM-based classifier, as depicted in Figure 2 [23]. An input feature to the BLSTM-based classifier was the 54-dimensional bottleneck feature that is identical to the output of the AE encoder, as illustrated in Figure 1. The BLSTM-based classifier comprises an FC layer with a dimension of (54, 128) and a BLSTM with 128 cells, followed by three FC layers with the dimensions of (256, 128), (128, 64), and (64, 2) each. Then, a max-pooling layer is applied to the outputs of the last FC layer, and the target for the max-pooling layer is a one-hot vector that represents ASD or TD for a given eGeMAPS feature vector. We consider this procedure to be a fine-tuning approach because the second module, the BLSTM-based classifier, is only trained using the bottleneck features that are already trained in the AE training framework. We refer to the model trained using this fine-tuning approach as the AE-BLSTM-FT model in this paper.

**Figure 3:**
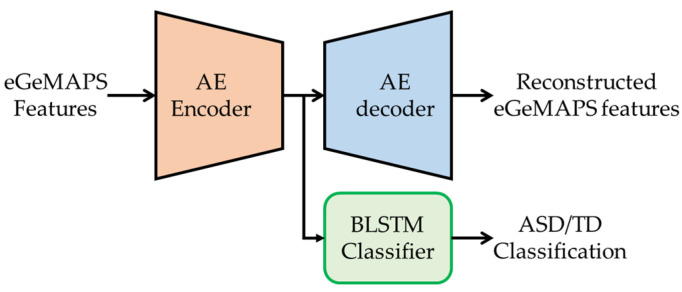
Network architecture for AE-BLSTM-based classifier for ASD/TD classification for joint training of AE encoder and BLSTM classifier, which we refer to as AE-BLSTM-JT.

Alternatively, we can consider the two modules to be a jointly trained pipeline, as illustrated in Figure 3, which we refer to as the AE-BLSTM-JT model in this paper. The AE-BLSTM-JT model is also trained using the combined loss, which we can define as follows:

(2) $$L=\alpha_{t}\cdot L_{recon}+\left(1-\alpha_{t}\right)\cdot L_{clsf}$$

where t is the training epoch, and $\alpha_{t}=\exp\left(-0.05t\right)$. Compared with that in Equation (1), the combined loss in Equation (2) is different given the weighting parameter, $\alpha_{t}$, according to the training epoch. By applying this equation, the AE feature extractor is primarily trained in the early stage of the training, whereas the BLSTM-based classifier is trained with more weight in the later epochs. Consequently, we evaluated two classifiers: AE-BLSTM-FT and AE-BLSTM-JT, which we trained using the eGeMAPS from the ASD/TD children’s voices and ASD/TD class labels, as described in Section 2.

## 4. Proposed End-to-End ASD/TD Classification Based on Pretrained Model

We must simultaneously consider both the short- and long-term features of the speech signals for the classification [26]. Usually, we extract the short-term features by applying feature-extraction techniques to each frame of the speech signal. Then, we can extract the long-term features by averaging the short-term features over a longer period of time. For example, the eGeMAPS includes both parameter types: short-term features, such as pitches, jitters, and formants, and long-term features, such as the mean lengths and standard deviations of the voiced/unvoiced regions [9].

Therefore, we first extracted 88-dimensional eGeMAPS features per frame, which corresponded to the short-term features. Then, we input these features into the AE to extract the bottleneck features, which were still short-term features because the AE operates in a frame-wise fashion. In contrast, the recurrent structure in the BLSTM classifier represents the long-term characteristics of the speech signals.

However, there is an ongoing question as to the extent to which a predefined feature set, such as the eGeMAPS, is beneficial to classification. Thus, we must determine the best predefined feature set for a given classification task. Recently, researchers have been directly studying neural network models using raw forms of speech signals for many speech-processing tasks, such as speech recognition and synthesis, and they have achieved better performances than those that use predefined feature sets. In other words, they use the spectrogram or the speech waveform itself instead of mel-cepstral coefficients [27,28,29]. The neural network model is called the E2E model because it directly uses raw forms of speech in the time or frequency domain.

Among these models, researchers have proposed the pretrained wav2vec2.0 model as a feature extractor [19]. The pretrained wav2vec2.0 model is a follow-up model of the wav2vec and VQ-wav2vec models [30,31], which can learn a representation of the raw waveform without labeled phonemes or graphemes. Researchers widely employ the model as a pretrained model in audio- and speech-processing tasks [32,33,34], as it has the advantage of from them from having to select the best predefined feature set task by task. In addition, the pretrained model usually comprises numerous parameters and is a priori trained with many speech and audio datasets, without regard to a specific task. Consequently, we can effectively apply the pretrained model in various downstream tasks with fine-tuning, such as automatic speech recognition, emotion classification, and speaker identification [28,29,32,33,34].

**Figure 4:**
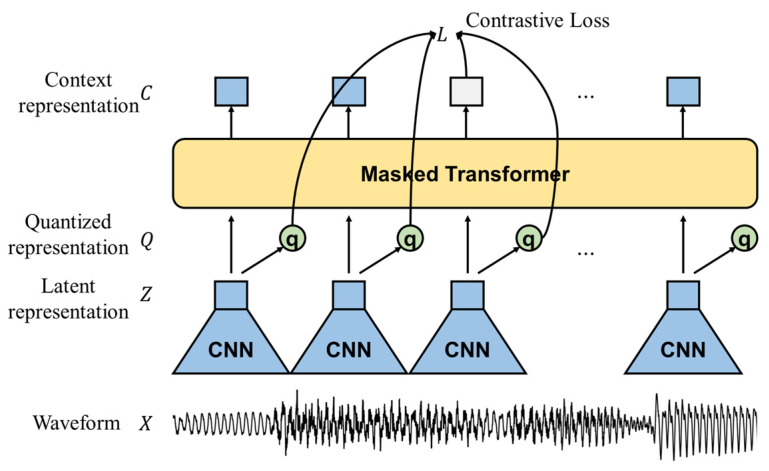
Network architecture for wav2vec2.0 and its pretraining stage based on contrastive loss.

We depict the network architecture of the wav2vec2.0 model and its pretraining stage based on contrastive loss in Figure 4 [19]. As demonstrated in the figure, the model comprises three parts: A multilayer convolutional encoder takes a raw waveform, X, and encodes it into a latent representation, Z. The transformer blocks [35] take the latent representation and build a contextualized representation, C. In parallel, the quantization module discretizes the latent representation, Q, and the conceptual representation is compared with the quantized latent representation to compute the total loss, L.

We can compute the total loss that comprises the contrastive loss, $L_{m},$ and diversity loss, $L_{d},$ with a weight, α, as follows:

(3) $$L=L_{m}+\alpha L_{d}.$$

We define the contrastive loss as the ratio of the cosine similarity, $sim\left(a,b\right)=a^{T}b/\left(|\left|a\right||\cdot |\left|b\right||\right)$, as follows:

(4) $$L_{m}=-\log\frac{\exp\left(sim\left(c_{t},q_{t}\right)/\kappa \right)}{\sum_{\tilde{q}\sim Q_{t}}\exp\left(sim\left(c_{t},\tilde{q}\right)/\kappa \right)}$$

so that the similarity of the context vector at time *t,* $c_{t}$, is compared with the quantized latent representation at time *t*, $q_{t},$ and with the $\tilde{q}$ from a distractor set, $Q_{t},$ with the $q_{t}$ and *K* candidates that are randomly selected at time *t*. In Equation (4), κ corresponds to a temperature parameter in the Gumbel softmax [36].

In addition, the quantization module in the wav2vec2.0 model is modeled with the product quantization, which selects the quantization using concatenated representations from codebooks, where G product codebooks and V codewords exist per codebook. Then, because the contrastive loss uses up to *K* (<*V*) candidates, the diversity loss, $L_{d},$ is used to reliably update all the codewords by maximizing their entropies, defined as follows:

(5) $$L_{d}=\frac{1}{GV}\overset{G}{\underset{g=1}{\sum }}\overset{V}{\underset{v=1}{\sum }}{\bar{p}}_{g,v}\log{\bar{p}}_{g,v}$$

where ${\bar{p}}_{g,v}$ is the v-th codeword in the g-th codebook. For this study, G=2 and V=320, which we set to the default values given in [19].

Therefore, the wav2vec2.0 model has context representation that is learned from finite and discretized representations with raw waveforms without supervised labels. In this sense, similar speech segments have closer descriptions, whereas speech segments with diverse characteristics are mapped to distant descriptions, which results in better speech segment classification if the context vectors are used.

**Figure 5:**
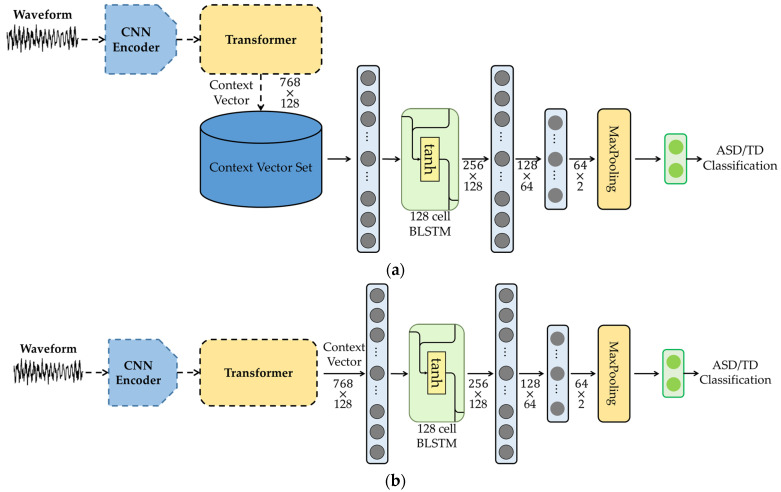
Network architecture for the proposed BLSTM-based classifier using wav2vec2.0 as pretrained model: (**a**) fine-tuning approach and (**b**) joint-training approach.

We present the network architecture of the proposed BLSTM classifier using wav2vec2.0, which we refer to as the W2V-BLSTM model in this paper, in Figure 5a,b. Similar to the AE-BLSTM-FT and AE-BLSTM-JT models, the proposed W2V-BLSTM model has two versions: W2V-BLSTM-FT and W2V-BLSTM-JT. For W2V-BLSTM-FT, a BLSTM-based classifier is fine-tuned with the downstream task of classifying the speech segments into ASD and TD. In particular, the quantization process is removed in the wav2vec2.0 model, and the context representations are only obtained from the input signal. We input the context representations into a BLSTM-based classifier to discriminate between the ASD and TD classes, as illustrated in Figure 5a. In other words, we extracted the context representation at time *t*, $c_{t},$ for a given waveform, and we only used the $c_{t}$ values to train the BLSTM-based classifier. In contrast, we obtained the W2V-BLSTM-JT model by jointly training all the parameters, including the wav2vec2.0 and BLSTM-based classifier.

The wav2vec2.0 model used in this paper is the base model described in [19]. As indicated in Figure 4, the first convolutional neural network (CNN) layer of the wav2vec2.0 model takes 400 samples for each input frame, which corresponds to 25 ms at a sampling rate of 16 kHz. Then, it applies seven convolutional layers to the input samples with different kernel sizes of (10, 3, 3, 3, 3, 2, 2) for each layer, where the strides applied to each kernel are set to (5, 2, 2, 2, 2, 2, 2), with a channel number of 512 each. The outputs of the CNN layers are projected into 768-dimensional vectors using the last FC layer of the CNN encoder module. Then, these vectors are transferred to the transformer module that consists of 12 transformer blocks. Each transformer block with eight multihead attention mechanisms processes the presentation with 768 input and output dimensions each, where the feedforward network in each transformer has the dimension of 3072 [19].

For the W2V-BLSTM-FT and W2V-BLSTM-JT models, we set the target vector for each speech segment as a two-dimensional one-hot vector that represents ASD or TD. We constructed the BLSTM-based classifier with the same structure as the AE-BLSTM classifier described in Section 3. We describe the performance evaluation in the next section.

## 5. Experiments

In this section, we explain the experimental setup used for evaluating the proposed ASD/TD classification models, including the dataset preparation and training procedure, with the hyperparameter settings of the models. Then, we describe the performance evaluation measures and discuss their results.

### 5.1. Experimental Setup

To train and evaluate the ASD/TD classifiers, we divided all the collected data described in Section 2 into three datasets: training, validation, and evaluation, with a ratio of 8:1:1. We applied the best-efforts arrangement so that the ages and genders were equally distributed among the three datasets. Afterward, we preprocessed each speech segment in the three datasets in two ways to the extract the features for the ASD and TD classification.

We performed the first preprocessing procedure to extract the eGeMAPS features by following the same procedure as in [23]. In other words, we divided each audio segment into 25 ms frames with a 10 ms overlap, and we applied the large-space extraction (OpenSMILE) toolkit [25] to each frame, which resulted in 88 different features of the eGeMAPS per frame. Next, we applied the mean-variance normalization technique to the eGeMAPS features, for which we acquired the normalization scaling from the training data and fixed it during the model inference. We used these normalized eGeMAPS features as the input features for the AE with the paired ASD/TD classes that corresponded to the diagnostic results of the speaker.

We performed the second preprocessing procedure as the input to the pretrained wav2vec2.0 model. First, we divided each speech segment into frames, and we set the frame size and overlap length identical to those of the first preprocessing procedure. However, instead of eGeMAPS features, we directly used the 400 samples per frame, which corresponded to 25 ms at a sampling rate of 16 kHz, as the input features for the wav2vec2.0 for the W2V-BLSTM-based classifiers.

We trained all the models, including the AE-BLSTM-based and W2V-BLSTM-based classifiers, using the Adam optimizer. As a learning rate scheduler, we applied an exponential learning rate decaying strategy with a coefficient of 0.9 after setting the initial learning rate to 0.001. We managed the training procedures for all the classifiers using the early stopping rule [37], which terminates the model training by detecting the minimized validation error with a 10-epoch patience. We took the wav2vec2.0 model used for the W2V-BLSTM-based classifiers from the base model already trained using 960 h of LibriSpeech data [38]. We implemented all the training and optimization approaches in Python 3.8.8 with PyTorch 1.12 [39], and we conducted all the experiments on an Intel(R) Xeon(R) CPU E5-2623 version 3 with a 3.00 GHz clock speed and an NVIDIA TITAN X Pascal architecture GPU. The source code for each model is available at https://github.com/AiTeRLab-GIST/E2E_ASD_DETECTION (accessed on 9 December 2022).

### 5.2. Performance Measure

We evaluated the performance of each model through the evaluation set, for which we equivalently sampled 2022 utterances comprising 1095 ASD utterances and 927 TD utterances from all age ranges for the overall estimation of the diverse vocal data. The compared models were as follows:

- A BLSTM-based classifier using only eGeMAPS features;
- A fine-tuned BLSTM-based classifier using the bottleneck features extracted from the eGeMAPS features (AE-BLSTM-FT);
- A jointly trained BLSTM-based classifier combined with an AE using eGeMAPS features (AE-BLSTM-JT);
- A fine-tuned BLSTM-based classifier using the wav2vec2.0 context representation features extracted from speech waveforms (W2V-BLSTM-FT);
- A jointly trained BLSTM-based classifier combined with the wav2vec2.0 model using speech waveforms (W2V-BLSTM-JT).

To measure the performance of each classifier, we converted the softmax output for each speech frame into a binary decision value of 0 or 1 for TD or ASD, respectively. If the average binary value over all the frames of an utterance was over 0.5, then we considered the utterance to be that of a child with ASD. We scored the performances using the means of the accuracy, precision, recall, and F1-score. We calculated each metric as follows: (1) we defined the accuracy as the number of correct decisions for both the ASD and TD samples over the total number of decisions; (2) we defined the precision as the number of correct decisions for ASD samples over the number of all the decisions answered as ASD; (3) we defined the recall as the number of correct decisions for ASD samples over the total number of ASD speech segments; (4) we defined the F1-score as the harmonic average of the precision and recall. In addition, we defined the unweighted average recall (UAR) as the average value for the ASD recall and TD recall, which was chosen in the Interspeech 2009 Emotion challenge to consider imbalanced classes [40]. We can more briefly present the metrics with equations, as follows:

(6) $$\mathrm{Accuracy}=\frac{\mathrm{TP}+\mathrm{TN}}{\mathrm{TP}+\mathrm{TN}+\mathrm{FP}+\mathrm{FN}}\;,$$

(7) $$\mathrm{Precision}=\frac{\mathrm{TP}}{\mathrm{TP}+\mathrm{FP}}\;,$$

(8) $$\mathrm{Recall}=\frac{\mathrm{TP}}{\mathrm{TP}+\mathrm{FN}}\;,$$

(9) $$F1-\mathrm{score}=2\times \frac{\mathrm{Precision}\times \mathrm{Recall}}{\mathrm{Precision}+\mathrm{Recall}}\;,$$

(10) $$\mathrm{UAR}=\frac{1}{2}\left(\frac{\mathrm{TP}}{\mathrm{TP}+\mathrm{FN}}+\frac{\mathrm{TN}}{\mathrm{TN}+\mathrm{FP}}\right)$$

where TP is the number of ASD decisions for a given ASD speech segment, FP is the number of ASD decisions for a given TD speech segment, TN is the number of TD decisions for a given TD speech segment, and FN is the number of TD decisions for a given ASD speech segment.

### 5.3. Performance Evaluation

First, we counted the number of model parameters for each model, which were 0.31 M, 0.33 M, and 5.75 M for the BLSTM-based, AE-BLSM-based, and W2V-BLSTM-based models, respectively. In this study, we constructed the two different fine-tuned and jointly optimized models from the same model architecture; thus, the AE-BLSTM-FT and AE-BLSTM-JT models had the same number of model parameters. Similarly, the number of model parameters for the W2V-BLSTM-FT model was identical to that of the W2V-BLSTM-JT model. The reason that the W2V-BLSTM model was heavier than the BLSTM or AE-BLSTM models was because of the pretrained model; however, we confirmed that the ASD detection was performed in real time on the hardware specification described in Section 5.1.

We compare the performance measures of the conventional classifiers using eGeMAPS features, such as the BLSTM, AE-BLSTM-FT, and AT-BLSTM-JT classifiers, in Table 2. As listed in the table, the BLSTM classifier exhibited the highest precision but had the lowest F1-score among all the conventional classifiers, which was caused by the biased decision boundary of the BLSTM; thus, most of the speech segments were classified as TD. In other words, the BLSTM classifier resulted in a smaller number of false-positive decisions, while it generated a large number of false-negative decisions from ASD to TD. For the AE-BLSTM-FT model, the F1-score was higher than the BLSTM because the number of false-negative decisions was substantially decreased. However, the accuracy and precision of the AE-BLSTM-FT classifier were lower than those of the BLSTM classifier, which implied that the AE-BLSTM-FT classifier had more true-positive and false-positive decisions than the BLSTM classifier.

In contrast, the two AE-BLSTM classifiers improved the F1-score, compared with that of the BLSTM classifier, which was because the AE contributed to the provision if more distinct features between ASD and TD than the eGeMAPS features; thus, the decision boundary was adjusted to evenly match ASD and TD. Finally, the table revealed that the AE-BLSTM-JT classifier achieved the best UAR because the joint training of the AE and BLSTM caused the adjustment of the bottleneck features and model parameters of the BLSTM to support the single goal of ASD/TD classification.

We compare the performance measures of the proposed W2V-BLSTM-FT and W2V-BLSTM-JT classifiers using speech waveforms as the input features in Table 3. Compared with the results in Table 2, the proposed W2V-BLSTM-FT classifier performed better in all the measures than the AE-BLSTM-JT using eGeMAPS features, which is because the pretrained wav2vec2.0 model implicitly extracted the critical features for the ASD/TD classification, in contrast to the eGeMAPS, for which the feature extraction is based on the deterministic approach. In other words, data manipulation in an E2E manner benefits this ASD/TD classification, as researchers have reported in other tasks [28,29,32,33,34].

Finally, we compared the performance of the jointly trained classifier with that of the fine-tuned classifier with the combination of the pretrained wav2vec2.0 model and BLSTM-based classifier. As revealed in the table, the W2V-BLSTM-JT classifier had a higher accuracy and precision than the W2V-BLSTM-FT classifier, which is because the wav2vec2.0 model was overfit to the training data, which resulted in the biased decision boundary between ASD and TD, similar to in the BLSTM classifier. For example, we trained the pretrained wav2vec2.0 model using 960 h of data; however, the training data in this work comprised around 3.21 h. Consequently, the W2V-BLSTM-JT classifier had a lower F1-score and UAR than the W2V-BLSTM-FT classifier because the insufficiency of the training data reduced the wav2vec2.0 capability.

## 6. Conclusions

In this paper, we propose an E2E model that is based on the pretrained wav2vec2.0 model to classify children with ASD and TD through their voices. The proposed E2E model comprises a wav2vec2.0-based feature-extraction model and BLSTM-based classifier. We trained the E2E model in two ways: (1) fine-tuning and (2) joint training. For the fine-tuned E2E model (W2V-BLSTM-FT), we directly used the context representation vectors of the wav2vec2.0 model for training the BLSTM-based classifier. In contrast, we constructed the proposed joint-training E2E model (W2V-BLSTM-JT) by concatenating the architectures of the wav2vec2.0 and BLSTM-based classifier. We trained the entire architecture together using the classification loss of the classified ASD and TD.

We compared the performance of the proposed E2E model with the conventional approaches based on an AE combined with the BLSTM-based classifier. Specifically, for the conventional fine-tuned model (AE-BLSTM-FT), we first trained the AE by the multi-task learning method, which combines the reconstruction and classification losses. Then, we used the bottleneck features from the AE to optimize the BLSTM-based classifier. In contrast, we trained the conventional joint-training model (AE-BLSTM-JT) as a pipeline using the combination of the AE and classification losses of the BLSTM-based classifier.

We evaluated the performances with the BLSTM classifier with the eGeMAPS input, and according to the results, the proposed method had the most accurate UAR results, considering both classes. In this paper, we highlight the feasibility of a pretrained model-based E2E classifier using a raw waveform with a wav2vec2.0 model. The effectiveness of the proposed approach at separating auditory features based on contrastive learning in the latent feature domain resulted in highly satisfactory ASD and TD classifications compared with the conventional models.

Consequently, the fine-tuned W2V-BLSTM-JT model had the highest F1-score and lowest UAR compared with the AE-BLSTM-based and W2V-BLSTM-FT-based classifiers. However, the performance reported in this paper was limited because we manually segmented all the prepared data. Thus, in future work, we will perform the following: (1) the automatic segmentation of the children’s speech; (2) the automatic separation of the children’s speech overlapped with other speech from parents or clinicians.

## Figures and Tables

**Table 1:** Distributions of age and gender (male/female) for subjects diagnosed with autism spectrum disorder (ASD) and typical development (TD).

Age (Months)	No. of Subjects Diagnosed with ASD (Male/Female)	No. of Subjects Diagnosed with TD (Male/Female)	No. of Child Subjects (Male/Female)
9–11	0/0	2/0	2/0
12–17	1/2	17/9	18/11
18–23	5/3	14/19	19/22
24–35	42/11	10/7	52/18
36–42	30/9	5/5	35/14
Average ± SD	32.20 ± 6.83	21.75 ± 8.17	27.39 ± 9.11

**Table 2:** Classification results of conventional classifiers using eGeMAPS features, such as BLSTM, AE-BLSTM-FT, and AT-BLSTM-JT classifiers.

	Classifier	BLSTM	AE-BLSTM-FT	AE-BLSTM-JT
Measure				
Accuracy		0.6400	0.6217	0.6474
Precision		0.6388	0.5714	0.6009
Recall		0.4941	0.6990	0.6872
F1-score		0.5572	0.6288	0.6412
UAR		0.6288	0.6276	0.6504

**Table 3:** Classification results of the proposed W2V-BLSTM-FT and W2V-BLSTM-JT classifiers using speech waveforms as input features.

	Classifier	Proposed W2V-BLSTM-FT	Proposed W2V-BLSTM-JT
Measure			
Accuracy		0.7077	0.7166
Precision		0.6757	0.7305
Recall		0.6969	0.6052
F1-score		0.6861	0.6619
UAR		0.7069	0.7081

## Data Availability

Not applicable.
